# Lecture capture affects student learning behaviour

**DOI:** 10.1002/2211-5463.13548

**Published:** 2023-01-16

**Authors:** Susanne Voelkel, Andy Bates, Terry Gleave, Carl Larsen, Elliott J. Stollar, Gemma Wattret, Luciane V. Mello

**Affiliations:** ^1^ School of Life Sciences University of Liverpool UK

**Keywords:** biological sciences, education, lecture podcast, lecture recording, lecture screencast, study behaviour

## Abstract

Lecture capture (the real‐time recording of live lectures) has become commonplace in higher education. It is popular with students who like the associated flexibility and believe that lecture recordings improve their grades. Here, we performed a survey (*n* = 694, 53% of the cohort) and set up focus groups (2 focus groups, 15 participants) to explore biological sciences students' perceptions of how lecture capture impacts their study behaviour when recordings are provided for every lecture and are made available to students without restriction. The participants in our study were convinced that lecture capture improved their learning, and many students noted that they were dependent on the recordings, thinking that without them, they would not be able to achieve good grades. Students reported that they spend a considerable amount of time watching recordings and making verbatim notes, leaving them little time for independent study. For many, lecture capture seems to reinforce the view that memorisation equals learning, a view that may be reinforced by knowledge‐focussed assessment formats. For most students, lecture capture did not affect self‐reported live lecture attendance patterns. However, about one‐third of the participants reported skipping more classes, and the same participants were more likely to postpone catching up on missed lectures. The outcomes of our study suggest that lecture capture provision may negatively affect some students' attendance and study behaviour, and thus, we suggest more needs to be done to mitigate against this.

## Graphical abstract

We asked students how lecture capture affects their study habits. Many respondents reported spending a lot of time watching recordings and making detailed notes. Most say that time for other study is diminished, but their attendance pattern is unaffected. Nevertheless, some skip lectures and may not catch up quickly, raising concerns about the negative impact of lecture capture for some.

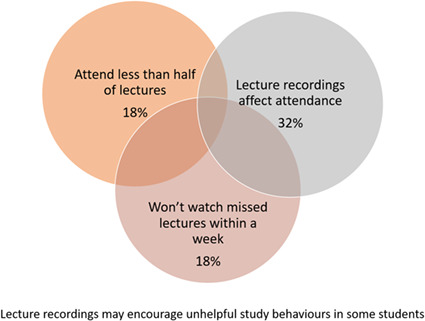

The term ‘lecture capture’ refers to the real‐time recording of live (face‐to‐face) lectures, simultaneously recording audio signals such as the instructor's voice as well as images (such as PowerPoint slides) and sound on the computer (here, the term ‘lecture capture’ does not include pre‐recorded lectures used for blended/online and/or flipped learning). Lecture capture is becoming increasingly commonplace in Higher Education settings in the UK [1] and worldwide [2]. According to O'Callaghan et al. [3] universities are under pressure from students to adopt web‐based lecture technologies such as lecture capture. In addition, factors such as high student numbers, inadequate funding and the increased need for students to work while studying may also play a role in an institution's decision to introduce systematic recordings of lectures [reviewed by 3, 4].

Many studies have investigated the benefits and drawbacks of lecture capture [reviewed by 5]. Dommett et al. [6] found that lecture capture reduces student anxiety by providing a supportive environment to some students. In addition, the technology plays a role in creating inclusive curricula, for example by helping to overcome language barriers [e.g. 7, 8, 9] and by supporting students with dyslexia and other specific learning disabilities [10, 11]. Undoubtedly, lecture capture is popular with students [e.g. 12, 13]. Students like the flexibility and convenience associated with the availability of lecture recordings [14]. They also report that lecture capture makes learning easier [15], for example by facilitating note taking and helping with revision [16, 17]. Many students perceive lecture capture as important to their studies [18] and think that it helps them to achieve better grades [19, 20, 21].

Whether lecture capture actually improves student learning and attainment is, however, still a matter of debate [reviewed by 5]. While some studies found a significant increase in student grades after introducing lecture capture [e.g. 22, 23], others reported no or only marginal effects [14, 24, 25]. In some cases, a decrease in academic performance was found [26]. There are a number of factors that could have an impact on whether or not lecture capture has a beneficial effect on attainment [27]. For example, Stroup et al. [21] report that low‐achieving students obtained significantly lower grades in a class that provided lecture capture compared with a class that did not, whereas high‐achieving students' grades were not affected. Artz et al. [28] found that the complexity of the material may also play a role. In a randomised control trial, the authors found that students tended to learn difficult concepts better when watching lecture recordings, but the reverse applied to easier topics.

A number of studies found that teaching staff appreciate many of the benefits of lecture capture to students [reviewed by 3]. In addition, lecture capture can have a positive effect on professional development by allowing lecturers to reflect on their teaching practice [29]. On the other hand, staff also report concerns about the potentially negative impact on student attendance in live classes [e.g. 30, 31, 32, 33]. Previous studies on the effect of lecture capture on attendance have shown conflicting results. Although several studies found no or only small effects [24, 34, 35], others reported a substantial drop in lecture attendance when lecture capture was introduced [12, 36, 37, 38].

If the introduction of lecture capture leads to increased absenteeism, this could potentially have a negative impact on student performance. Many past studies have demonstrated that class attendance is positively correlated with student attainment [39, 40, 41]. Although most of these studies were undertaken in the absence of lecture capture, others have found that lecture capture is no substitute for lecture attendance [37, 42], especially for low‐achieving students [43, 44]. This would indicate that, even if lecture capture is being provided, absenteeism could still be detrimental to some students, in particular, if they use it to substitute rather than to complement attendance.

## Rationale for this study

Many studies on lecture capture have been undertaken in settings where the technology was new and/or had only been introduced in a single course or section of a course. It is, however, important to evaluate the impact of lecture capture in a setting where it is routinely used. This allows insight into student perceptions and behaviour when they have come to expect being provided with a recording immediately after each live lecture, meaning it is the norm rather than the exception or novelty. At the University of Liverpool, all live (face‐to‐face) lectures have been recorded via the University's stream capture software since 2016. The full, unaltered lecture recordings are then made available to all relevant students via the University's virtual learning environment within a few days of each live lecture, and they remain available until the end of the academic year. Taking a mixed method approach, the aim of this study was to evaluate how lecture capture affects student learning behaviour (including attendance) under these circumstances.

## Methods

This study focusses on the recording of traditional, didactic live lectures, which may or may not include a small proportion of student interaction in the form of quizzes, questions or polls. Ethics approval was granted by the University of Liverpool Ethics Committee in December 2018. A mixed method approach was taken, using an ‘explanatory sequential design’ [45]. We started by collecting quantitative and qualitative data from a student questionnaire, followed by qualitative data from student focus groups. This approach was taken so that the questionnaire results would inform the focus group questions with the purpose of explaining and adding more depth to the qualitative data. All participants were provided with a participant information sheet and focus group participants signed an informed consent form before the start of the meeting.

### Questionnaires

**Fig. 1:**
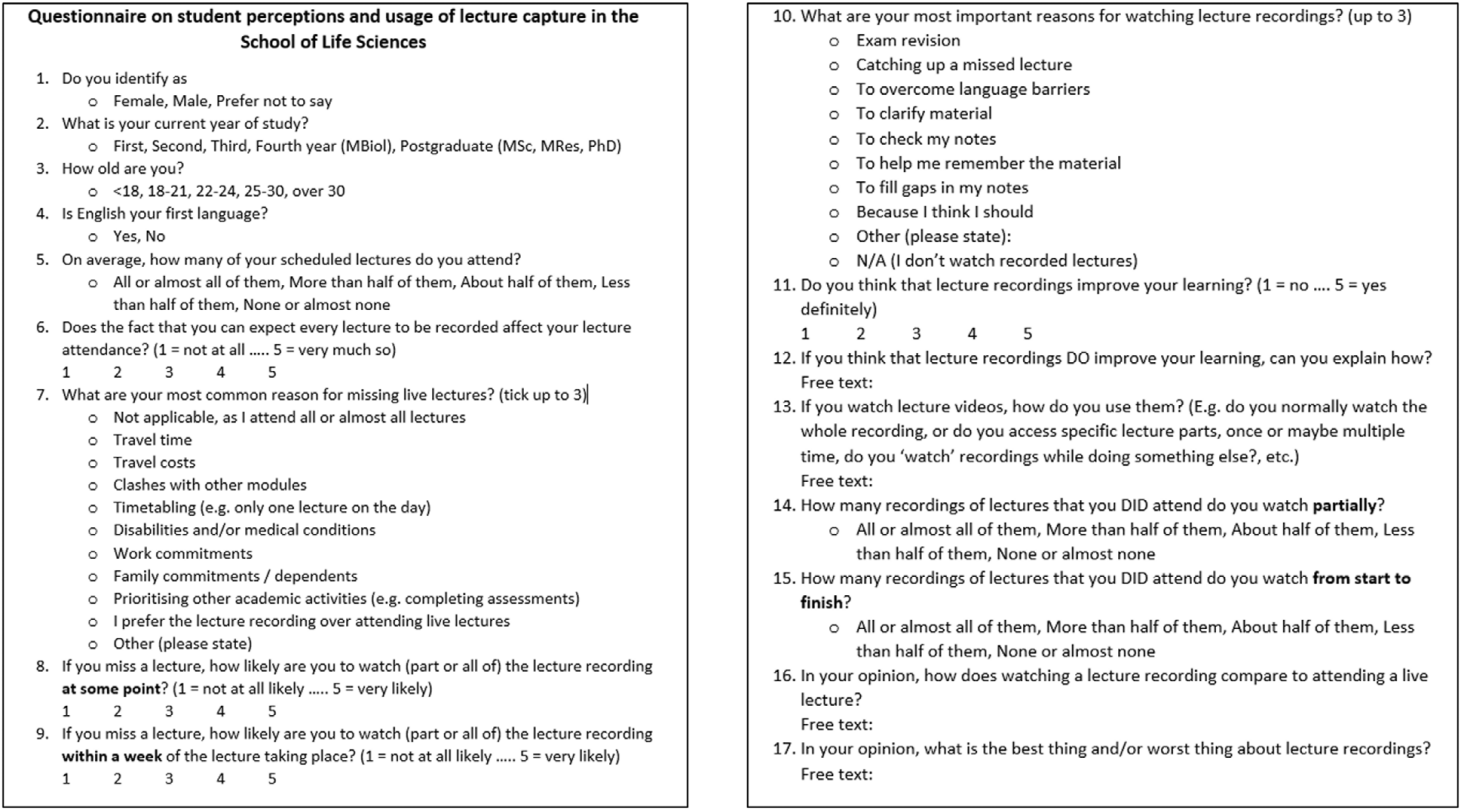
The questionnaire used in the study.

In March 2019, all students in the School of Life Sciences (Undergraduate Years 1–3, Postgraduate taught, appr. 1300 students) were invited by email to complete an anonymous questionnaire about their perceptions and usage of lecture capture and their lecture attendance. Questions included open and closed questions, some of which were Likert style (Fig. 1). The questionnaires were completed electronically.

### Focus groups

All students were invited by email to attend focus groups to discuss their perceptions and use of lecture capture. Two student focus groups were held in November and December 2019, respectively, and participants received a £10 voucher as an incentive. The focus groups were facilitated by staff from outside the School who did not teach or know the students; the participants remained anonymous. The focus groups, designed based on the questionnaire analysis, used a semi‐structured approach and the main aspects explored were lecture recordings usage and their impact on student's learning and attendance of live lectures. The main questions used were: (a) How do you use the lecture recordings (including ‘how do you choose which recording/which part you watch?’, ‘How much time do you spend watching recordings’, etc.), (b) How do lecture recordings affect your learning (including ‘what does learning mean for you?’) and (c) Does the fact that lectures are being recorded change your behaviour in lectures? (including ‘are you more tempted to switch off during lectures? Does it inhibit you asking questions?’). All 4 years of study were represented. The first focus group meeting had seven participants (two male, five female) and lasted 56 min, the second had six participants (three male, three female) and took 29 min. The meetings were audio recorded and transcribed independently. Transcripts were sent to the participants of each meeting to check, and all agreed that they were accurate.

### Data analysis

The responses to open questions in the questionnaire and the transcripts of the focus group recordings were analysed using thematic analysis [46]. The focus group transcripts were analysed independently by two authors (SV and LVM) who then agreed on the main themes. Questionnaire comments were coded independently by the other authors who then together cross‐checked and agreed the themes. Individual comments could be associated with one, more than one or none of the main themes, resulting in the appropriate number of ‘codings’.

Quantitative questionnaire data were analysed using spss statistics software (version 26, IBM Corp., Armonk, NY, USA). To analyse correlations, Spearman's Rank Order Correlation was used. For comparisons between groups, the Kruskal‐Wallis test was used, followed by *post hoc* Mann–Whitney *U* tests between pairs with Bonferroni adjustment to control for type I errors. Statistical significance was accepted at *P* < 0.05 level.

## Results

### Student questionnaire results

694 students completed the questionnaire, which is a response rate of approximately 53%. 69% of the respondents were female and 30% male (1% did not provide this information), and 81% declared English to be their first language. The demographics of the respondents were representative of the whole student cohort.

#### Lecture attendance

**Fig. 2:**
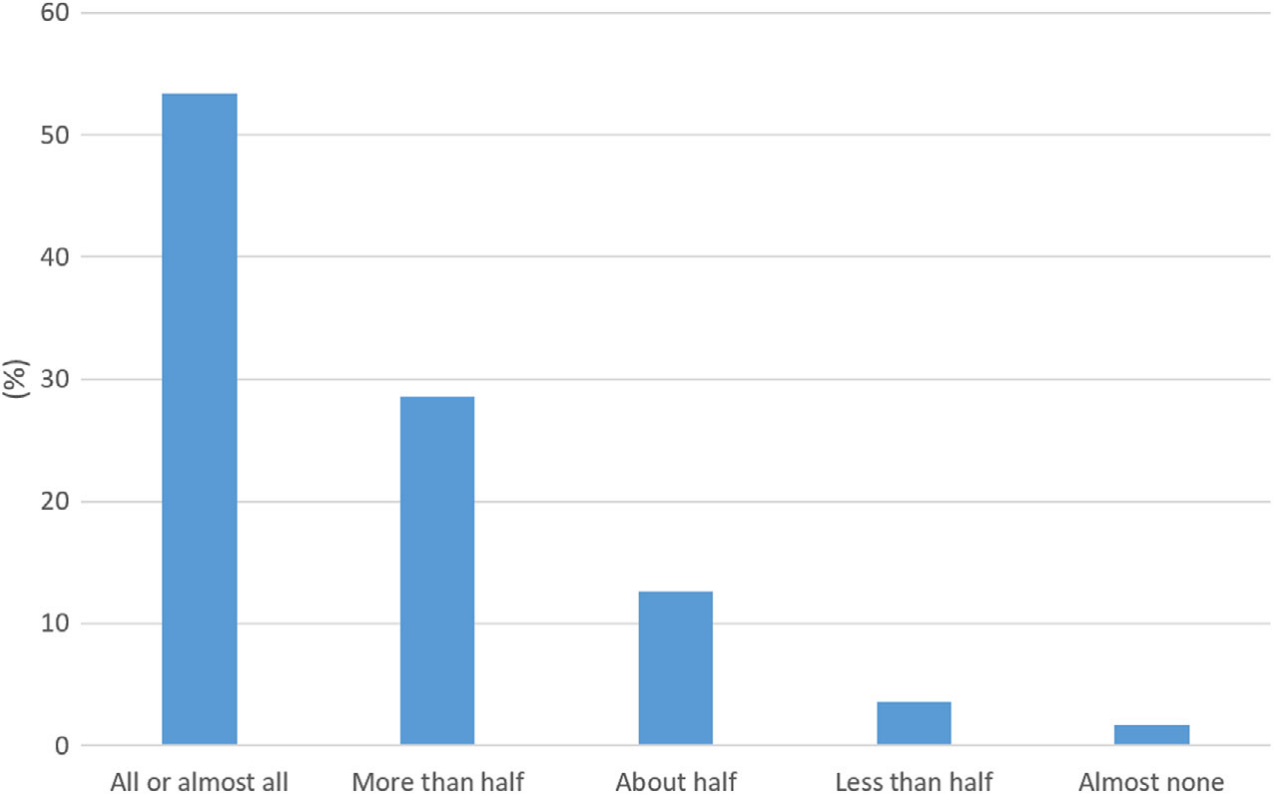
Student responses to the question ‘On average, how many of your scheduled live lectures do you attend?’ (*N* = 687).

82% of the participants reported to attend on average more than half of their live lectures, with 53% saying they attended all or almost all of them (Fig. 2). 5% said they attended less than half of the lectures, with almost 2% attending none or almost none. A Kruskal–Wallis test revealed statistically significant differences in self‐reported attendance between year groups (Yr1, *n* = 221; Yr2, *n* = 289, Yr3, *n* = 145, Yr4, *n* = 32), Χ^2^ (4, *n* = 687) = 14.776, *P* = 0.005. Year 2 students reported a higher median score (Md = 2, meaning they reported attending ‘more than half’ of the lectures) than the other groups, which all reported a median value of 1 (meaning attending ‘all or almost all’ lectures). A Mann–Whitney *U* test showed there was a significant difference in reported attendance between year 1 and year 2 (*U* = 26,689, *z* = −3.51, *P* < 0.01) but not between year 2 and year 3 (*U* = 20,258, *z* = −0.61, *P* = 0.542). There was no significant difference between self‐reported attendance of students with English as first language and students whose first language is not English (*U* = 34,559, *z* = −0.958, *P* > 0.05).

**Fig. 3:**
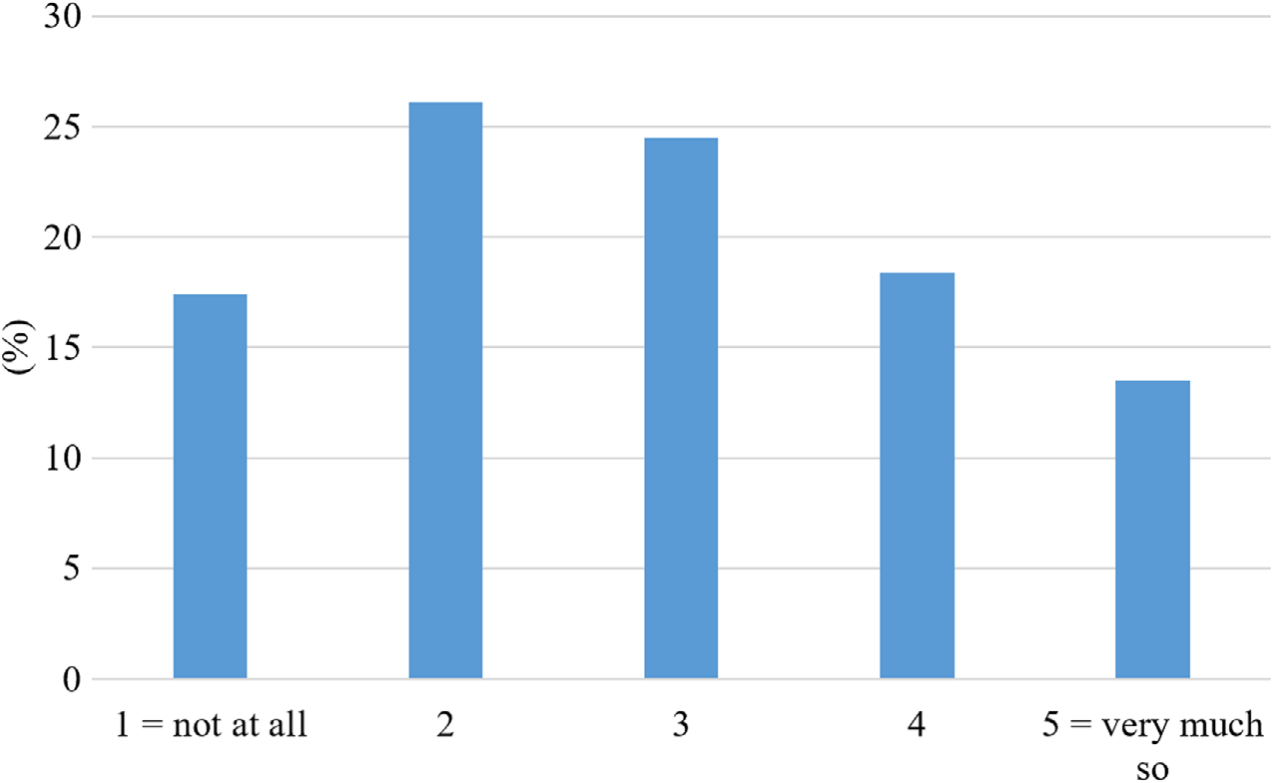
Student responses to the question ‘Does the fact that you can expect every lecture to be recorded affect your lecture attendance?’ In this Likert‐style question, students were asked to indicate their answer on a scale from 1 to 5, with only the extremes identified as 1 = Not at all, 5 = Very much so (*N* = 685).

Students were then asked whether the fact that they can expect every lecture to be recorded affected their lecture attendance. 32% of the respondents indicated that lecture recordings did affect their attendance, with 14% choosing ‘very much so’ as an answer (Fig. 3). There was a statistically significant, medium size relationship between the extent to which lecture capture provision affected students' attendance and their self‐reported attendance rates (Spearman rho = 0.39, *P* < 0.01, coefficient of variation = 15.5%). Students who reported that lecture capture provision had little or no effect on their lecture attendance were more likely to report high attendance rates and vice versa. A Kruskal–Wallis test indicated statistically significant differences in the extent to which different year groups reported to be affected by lecture capture (Yr1, *n* = 191; Yr2, *n* = 290, Yr3, *n* = 145, Yr4, *n* = 33), Χ^2^ (4, *n* = 687) = 15.586, *P* = 0.002. Year 1 students reported a lower impact on their lecture attendance (Md = 2) than the other groups (Md = 3) (see Fig. 3 legend for explanation). The difference was statistically significant between year 1 and year 2 (Mann–Whitney *U* test, *U* = 26,165, *z* = −3.49, *P* < 0.001) and between year 1 and year 3 (*U* = 12,957, *z* = −3.05, *P* = 0.002).

#### Usage of lecture recordings

**Fig. 4:**
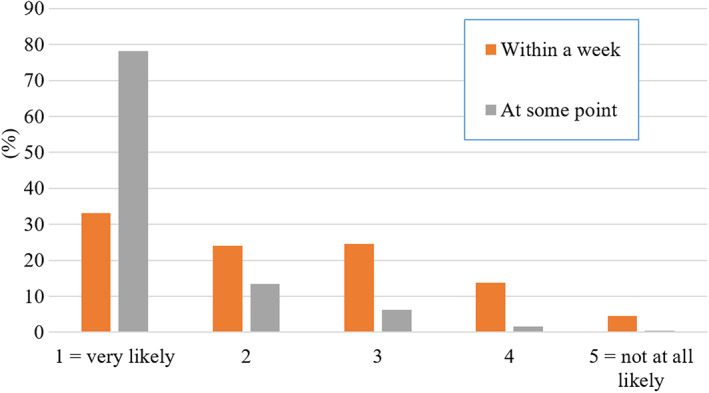
Student responses to the question ‘If you miss a lecture, how likely are you to watch the lecture recording… within a week/ at some point’. In this Likert‐style question, students were asked to indicate their answer on a scale from 1 to 5, with only the extremes identified as 1 = Very likely, 5 = Not at all likely (*N* = 685).

**Fig. 5:**
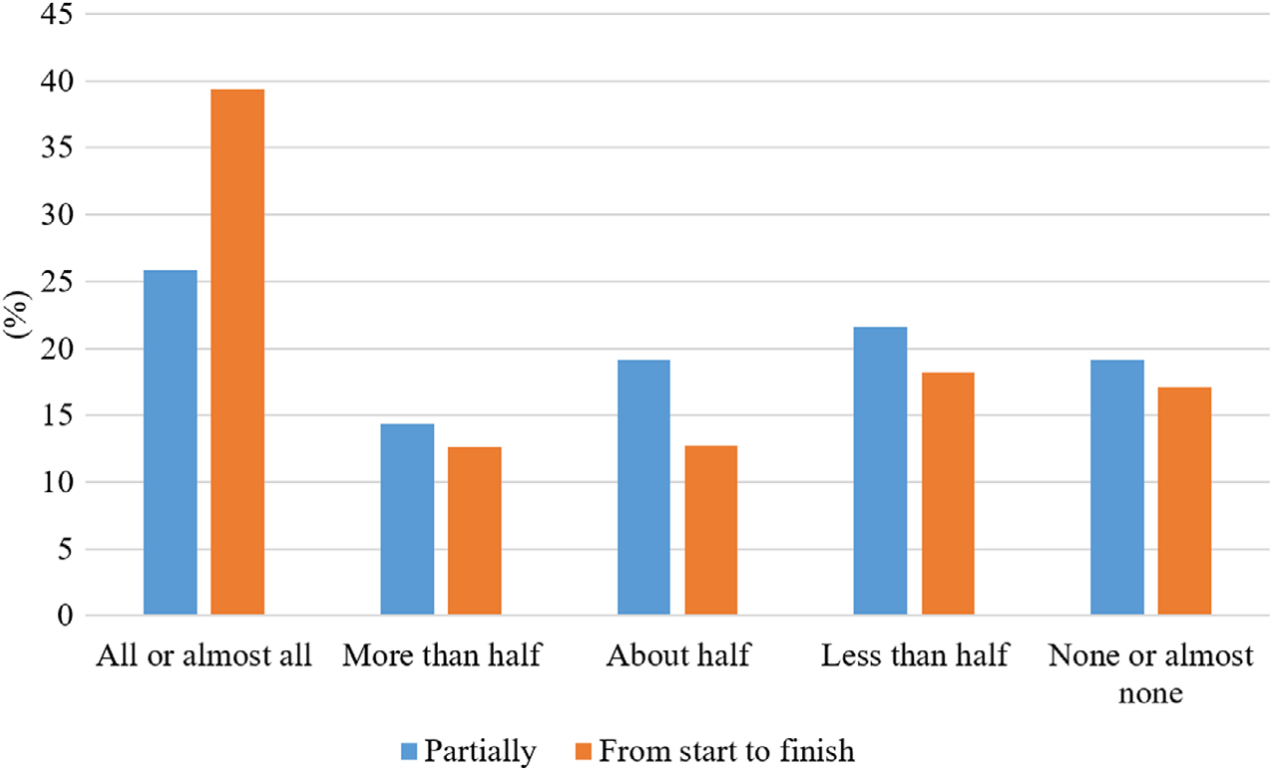
Student responses to the question ‘How many recordings of lectures that you DID attend do you watch… partially/ from start to finish’ (*N* = 685).

Students were asked how likely they were to watch recordings from lectures that they had missed. While 78% said they were ‘very likely’ to watch the recording at some point, only 33% were ‘very likely’ to watch it within a week of the missed lecture (Fig. 4). There was a significant but weak correlation between self‐reported attendance and watching missed lectures within a week (Spearman rho 0.26, *P* < 0.01). The higher the reported attendance, the higher the self‐reported likelihood that a student watched a missed lecture within a week. When asked how many of the attended live lectures they would also watch as recordings, 26% of the students said they would watch ‘all or almost all’ recordings partially, and 39% said they would watch ‘all or almost all’ from start to finish (Fig. 5).

**Fig. 6:**
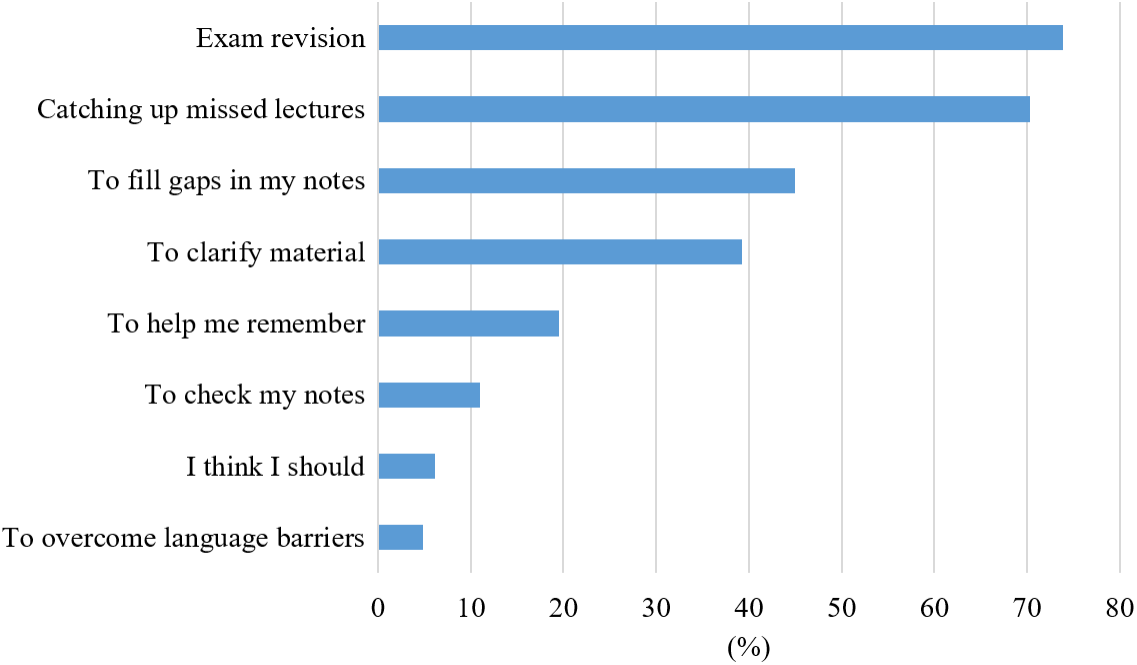
Students' answers to the question ‘What are your most common reasons for watching lecture recordings? Choose up to three’.

Students were then asked to choose their most common reasons for watching lecture recordings. The most popular reasons were exam revision, catching up on missed lectures, filling gaps in their notes and clarifying material (Fig. 6). When asked to explain in more detail how they used the lecture recordings, many students said they would watch the whole lecture, in particular (but not only) when they had missed the lecture (Table 1). Others said they would watch specific parts of the lecture, where there were gaps in their notes or where they struggled with the content. Some students said they would watch certain sections multiple times and others specifically referred to revision. An overarching subject was note taking: In most comments, students referred to making, completing, reviewing or editing their notes. Students often pause or adjust the speed of the recording to enable note taking.

**Table 1:** Responses to the open survey question ‘If you watch lecture recordings, how do you use them?’ 538 students provided a response, resulting in 1155 codings.

Themes	Subthemes	% of codings	Illustrative quotes
Mode of watching	Watching the whole recording	39	‘Watch recordings once, then go over them again whilst making lecture notes’. ‘Watch the whole thing if I missed the lecture’
	Watching specific parts	23	‘I'll skip to the part where I have a blank in my notes’. ‘Usually access different parts which I make notes during the lecture to go back to’
	Pausing, adjusting speed	15	‘I change the speed of the recording. When I'm taking notes I will slow down the speed of the speaker. When I'm re‐watching lectures before an exam I will increase the speed of the speaker’
	Watching multiple times	7	‘Sometimes I might listen even twice if I don't understand the lecture’. ‘[I] might watch sections multiple times’
Purpose of watching	Use for revision	9	‘Watch all for revision then watch sections if stuck when revising’
	Checking and editing notes	7	‘locate parts where notes are thin/don't make sense’

#### Lecture capture and learning

**Fig. 7:**
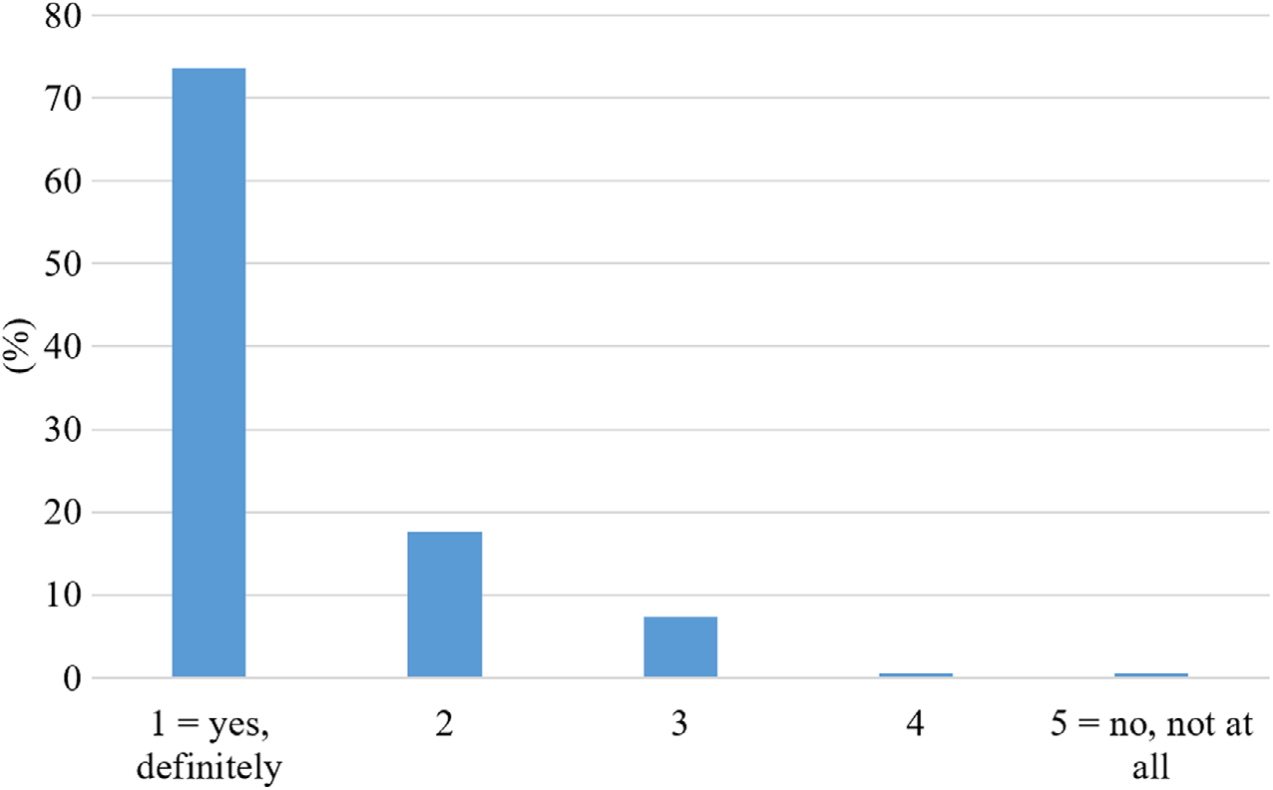
Student responses to the question ‘Do you think that lecture recordings improve your learning?’ In this Likert‐style question, students were asked to indicate their answer on a scale from 1 to 5, with only the extremes identified as 1 = Yes, definitely, 5 = No, not at all (*N* = 689).

Most students (74%) thought that lecture recordings ‘definitely’ improved their learning (Fig. 7). Only a small minority (1.5%) did not perceive lecture capture as beneficial for their learning. When asked to explain how lecture recordings improved their learning, written responses fell into two themes: the learning process itself and filling gaps (Table 2). For the first theme, a large proportion of respondents said that lecture recordings help them understand the content and to consolidate their learning. Secondly, they find it helpful to be able to learn at their own pace. Catching up on material that they missed during a live lecture was a common topic related to the second theme (filling gaps), as was the completion of notes, echoing the results from Table 1.

**Table 2:** Responses to the open survey question ‘If you think that lecture recordings do improve your learning, can you explain how?’ 529 respondents provided comments, which led to 1021 separate codings.

Themes	Subthemes	% of codings	Quotes
Learning process	Understanding and consolidation of learning	25%	‘Recap what you've already heard in a lecture. Sometimes hearing something for a second time makes it “click” and you understand it better’ ‘Helpful to look back at how the lecturer explained things’, ‘Enables me to go over parts of the lecture I didn't understand or parts that I wasn't concentrating [on]’
	Memorising	3%	‘Watching lectures back really helps me to remember facts’, ‘Means you can revisit the material and learn through repetition’
	Exam revision	9%	‘Allow me to have complete notes for revision for my exams’, ‘It also allows me to re‐watch around exams and make sure I fully understand’
	Own pace	17%	‘I can learn it a bit better when I can pause and rewind’, ‘They allow you to go at your own pace in an environment you are comfortable in when you are ready to cover the material’
Filling gaps	Completing notes	20%	‘I can add information to my notes that I may have missed during the lecture’, ‘Fill in any gaps in notes, clarify any confusion’
	Catching up information that was missed during the lecture	18%	‘Sometimes I can't catch up because the professor was talking too fast, the stream lectures remind me of what I missed’, ‘It can be hard to concentrate for the full 50 minutes sometimes and if I do zone out it's nice to have the ability to go through the recordings to fill in the parts that I missed’
	Catching up a missed lecture	5%	‘Always have it as a back‐up if I do not make it to the lecture’, ‘When I miss the class, I can watch it’
	Allows listening during lecture without note taking	3%	‘They allow me to not worry about taking complete notes during the lecture and pay more attention to what the lecturer is saying, and then use the recordings to complete my notes after the lecture’

#### Recordings versus live lecture

Slightly more students said that they preferred live lectures over lecture recordings than *vice versa* (13 *versus* 10%, Table 3). Reasons included more interactions with staff and other students (including the opportunity to ask questions) and better engagement/focus in live lectures. Some also mentioned technology‐related problems with recordings, such as bad sound quality or the fact that whiteboards and laser pointers were not recorded. On the other hand, many students liked the fact that they could watch lecture recordings at their own pace (including pausing and rewinding). Other advantages of lecture recordings included the opportunity to repeat sections and take better notes. Overall, many of those students who liked live lectures better than recordings also listed the benefits of the latter and would use them as an additional resource rather than a replacement.

**Table 3:** Responses to the survey question ‘In your opinion, how does watching a lecture recording compare to attending a live lecture?’ 540 respondents provided a comment, resulting in 1188 codings.

Themes	Subthemes	% of codings	Illustrative quotes
Preference	Live lectures are better than recordings	13	‘Doesn't compare. Live lecture better’, ‘Attending a live lecture is more beneficial’, ‘I do prefer the live lecture, and a recording will never be as good’
	Lecture recordings are better than live lectures	10	‘Sometimes it's better and you can understand more than when attending a live lecture itself’, ‘I much prefer a recording…’
	No difference	5	‘They're the same’
Engagement	Better engagement in live lectures	6	‘I concentrate better at a live lecture than at home as less distractions’, ‘The recorded lecture/stream isn't as engaging or interactive. It's easier to focus and pay more attention at live lectures’
	Can focus better on recordings	3	‘I concentrate more watching a lecture recording because I'm usually revising or trying to understand a topic better’, ‘There are less distractions in the library or at home’
Options	Interaction with staff or peers	7	‘If you need to ask questions the live lecture is better’, ‘There is also the opportunity to interact with the lecturers after the session, as well as other students during and after the lectures’
	Problems with recordings	5	‘A lot of the time the audio is tricky so you can't always hear everything that was said. [And] if the lecturer is gesturing to one particular part of a diagram or text then you are unable to know what they are referring to’
	Allows watching at own pace	23	‘Can allow students to pause the stream, to make sure they understand the content before progressing further’, ‘Watching a lecture recording is more useful as you can pause it to give you time to keep up’
	Ability to repeat/ recap	13	‘Easier to clarify hard subjects by rewinding multiple times’, ‘It's better as if I didn't understand a word of what the lecturer was saying, I could go back and listen again’
	Better notes	11	‘[Recording] allows you to make more detailed notes’, ‘It's easier to take notes as you can pause it and write down a point’, ‘Takes longer to watch the recording, but I get more detailed notes as a result’
Environment	Choose the time and environment	2	‘I prefer being in my own living space’, ‘Makes it available to watch/catch up in your own time’

### Student focus groups

The participants of the two focus groups all agreed that lecture capture was important for their learning (Table 4). The emotional impact of lecture capture was a strong theme: Many participants expressed a feeling of panic and stress at the thought of not having recordings. In turn, they found that lecture recordings can reduce stress during live lectures, being able to enjoy the lecture without having to worry about taking notes or missing something. The participants reported different approaches to how much of the recording and when they watch, with some watching the whole recording, others only specific parts, some watch throughout the semester and others only during revision time. However, there was general agreement that lecture recordings were very helpful if students missed a lecture or if they struggled with certain parts of an attended lecture. Note taking was an important aspect of the process, with recordings being essential for filling in gaps in the notes.

**Table 4:** Focus group themes. Numbers in brackets refer to individual participants. Participants 1–7 took part in the first focus group, 8–13 participated in the second focus group.

Theme	Sub theme	Quotes
Emotional effects of lecture recordings	Dependency	‘And, so, yeah, especially for fast paced lectures where you're like, you know, what was that? If I didn't have a stream capture, I wouldn't be able to understand any of it probably’. [3] ‘I wouldn't be able to do as well without lecture streams’ [8] ‘I couldn't imagine not having the stream capture’. [12] ‘A panicked feeling [if there is no recording]’ [3] ‘I do get stressed when it's not recording’ [1] ‘it's like really stressful […] if you miss it you've missed it, and that could affect your grade’ [3] ‘I feel like I would be quite stressed sitting in there knowing that was the only time I was going to hear the lecturer saying it’. [12]
	Reducing stress	‘Sometimes when you go to a lecture you have that tendency to make, you know, word by word what the lecturer has said rather than actually enjoying the lecture. So, the lecture capture gives you like an opportunity to enjoy the lecture, also making notes, and if you miss something out of the lecture, but not avoiding the enjoyment of the lecture’. [6] ‘Yeah, it makes you less stressed knowing that it's there’ [3]
How students use lecture recordings	Time spent on watching recordings	‘I usually go through [the recordings] like two or three times while we're revising’. [2] ‘Like even though it's like a 45 minute lecture it will take me like two hours to get through it.’ [1] ‘I spend a lot of hours doing stream captures, doing notes’. [9] ‘I spend around one quarter of my whole study time on stream capture’. [11]
	Targeted watching	And then if there's a bit that I haven't perhaps understood, I'll go over that bit. Or if the whole lecture was just—you know, I couldn't understand any of it, then I'll have to do the whole thing again and pause it like a hundred times. [3] I never really like watch a whole stream capture, it's more just like I'll write down the time of like a specific part that I just missed or like daydreamed over or if it's too fast. So, I tend to just like focus on that one bit and then don't watch the rest of it. [1]
		‘I usually watch the lectures that are like sort of like a complicated subject, so it's like something I'd need more explanation on and where I'd didn't really understand as much in the lecture’ [13]
	Timing of watching	‘I would keep up with it during the term and then I'd have all my notes ready and perfectly made, and then I'd revise from what I've understood. Like I wouldn't want to, when I'm revising, be like oh I've missed something, and then have to go to the stream capture when I'm in the middle of revising’. [1] ‘Like my percentage [of time] definitely goes up during revision time when I want to review the work that we've already done, but I won't use it an awful lot during the actual semester, I think’. [7] ‘During like term time I'll only watch lectures that I missed on stream capture, and then during like when revising I'll go back and just watch the lectures that I've found most difficult’. [12]
Purpose of watching	Revision	Yes, and for me it's like I'm actually going to the lecture capture straightaway, you know, it's ready for your revision [6] I feel like going [to watch] stream capture and like just understanding what the lecturer said word by word is probably going to be the revision. [13]
	Note taking and understanding	Because you can write it out in more detail if you have the lecture capture, so you can probably have done an overview during the lecture and then you can go back into more detail, because you can pause and go back and write word for word what they've said and it makes more sense. [4] Like in a way you'll understand it rather than rushing to write it down and then being like oh, I didn't actually understand any of that. [3] [without lecture capture] There would just be like loads of like gaps in my notes [1] When you watch the lecture back you're pausing it and writing notes and that's so much better than when you're in the lecture and like not understanding the concept and you go onto the next one. [9]
Behaviour in lectures	Note taking during lectures	I also take notes during the lecture, and I just use the stream capture to just make some additions [11] I do like all my notes in lectures handwritten, so if there's like a bit that I've missed or can't understand I just do like lots of question marks in the margin where I can come back to it. [10] In first year I made notes like by hand, as in wrote them out, so therefore I had to just re‐watch all of them because I was so slow at writing. And then the second year I started typing it and I was a lot better, and then just looked at sections of it. So yeah, I can't keep up if I'm just writing. [4]
	No change in behaviour	People don't like to ask questions [3] I wouldn't put my hand up if it was recording or not. [4] I wouldn't answer if it was being recorded or not recorded [12] No, I wouldn't say it affects my concentration because I'm concentrated on getting everything down and understanding it there so that I don't have to re‐watch it. Because I'll try and save myself time if I can so I'm focusing fully when I'm in there to try and make sure I don't need to re‐watch it. [12]
What affects use of recordings	Approach changes between year 1 and year 3	I used to re‐watch all of them, but now it's—now that I've gotten better, I don't, I just watch bits, so that's changed. [2] Definitely in first year, I felt like I had to go over everything, even if I'd made the notes, I was like oh, because I didn't know what the exams would be like, because I was very stressed about it. [3] And I kind of feel like now like in second year you do need to read more around like certain things […], not just […] listening to the stream lecture. [2] First year there weren't as many assignments so I had more time and I was like what can I do to be benefitting my studies, as sad as that is. […] like so I'd go back and watch them and made sure that my notes were like immaculately like thorough, whereas like now I think I've got more the hang of that I've like got to make not necessarily word for word notes, but more like key structured notes which I can do [8]
	Impact of assessment	It's like that at any point there could be something, one sentence come up that you could be tested on, so I'm just like I should probably watch them all [2] But in my first and second year I didn't do any extra reading of anything really, although they sort of suggest it in second year. And then in third year it becomes like mandatory to do it and there's like marks for showing that you've read something else, so you have to do it. [3] I think it depends on the module as well, the exam that you're doing, you know that it's going to be an essay, then they're going to look for outside reading and for you to reference that. Whereas if it's going to be like short answer questions that's probably going to be more specific to the lecture capture so I'm less likely to read around it [7] I think it's definitely different from first year to third year. Like in the first year people would definitely need to get all of the details because—because they were multiple choice questions it could be pretty much anything they've said [12] You're just kind of doing what you think is the best use of your time to get the best grade you can [8] It's like you're not necessarily focused on always understanding it, you're focused on what they're going to ask in the exam [12]
Using other resources	Time constraints	I mean, I could read textbooks but I'm still trying to catch up, so I don't really end up having time to look at textbooks [9] After going over [the recording] like for two hours, you can't then on top of that spend two hours looking at things that could be related to the topic as that could be anything [3] I don't really end up having time to look at textbooks [9] There's just not enough time in the day. [10]
	Convenience	I prefer listening to somebody talk [3] It takes far longer, I find, reading research papers than it does just listening to the lecture. [2] But it takes a long time to read around a subject when you could just put on lecture capture and watch that instead [4] How would you know you were reading the right thing? [3] […] if you read something then a lot of the time it's really difficult to read… It takes far longer reading research papers than it does just listening to the lecture. [2] Obviously it's just like the easiest thing to do is just to go to stream capture [1]

There was general agreement that recordings do not change the way students behave in lectures: Most of them still attempt to take notes and pay attention (although they sometimes lose concentration). The recordings also did not affect whether or not they would ask or answer questions during the lecture: Generally, students said they would not anyway.

There was an indication that the way students use lecture recordings may change throughout their studies: Some participants said that while in year 1 they tended to watch all lectures and attempted to create a word‐for‐word ‘transcript’ of the lecture for their notes, they would use the recordings more strategically in later years as they would focus more on concepts and may read a bit more. Assessments also appear to affect the way students use the recordings and other resources. Students would rely more on recordings when assessments are knowledge‐driven (such as multiple choice or short answer questions), whereas outside reading becomes more important for evaluative written pieces. But apart from assessment‐driven outside reading, students reported not reading textbooks, partly because of a lack of time (as watching lecture recordings takes a lot of time) and partly because they feel it is easier to listen to recordings. For some, watching lecture recordings amounts to revision and the recordings contain everything they need for their assignments.

## Discussion

This study explores students' perceptions of lecture capture when recordings are reliably being provided for every lecture and are available to students without restriction. What is more, University of Liverpool students have come to expect lecture capture since it became obligatory more than 3 years ago. Some of our results echo previous findings. For example, similar to other studies [e.g. 19, 20, 21] most students in our study reported that lecture capture improved their learning (equating learning with attainment). Similarly, when asked how they used lecture recordings, the answers of our participants mirrored previous studies [e.g. 17, 20, 38, 47] with the most frequent reasons for watching being exam revision, catching up missed lectures and filling in gaps in their notes. This indicates that popularity and purpose do not change when lecture capture becomes the norm rather than a novelty or an exception.

The main aim of our study was to evaluate if and how the provision of lecture capture affects student learning behaviours. One aspect of learning behaviour is the attendance (or otherwise) of live lectures. Almost half of the respondents in our study reported that they attended all or almost all of their live lectures, whereas almost 18% said they attended half of their lectures or fewer. About one‐third of the respondents also stated that the provision of lecture capture negatively affected their attendance. This is in line with other studies where students reported a drop in attendance when lecture recordings were introduced. Percentages range from 14% [48] to more than 55% of respondents reporting a negative impact on their attendance, with some students saying they stopped attending classes altogether [25].

There are many reasons why students may skip lectures, including caring responsibilities, the need to earn money, social anxiety, as well as physical disabilities and issues of neurodiversity [11]. In addition, some students in our study told us that they learn better from recordings than from attending live lectures. Therefore, absenteeism in itself does not have to be detrimental to student learning. Nordmann et al. [43] reported that in first‐year students, both attendance and the use of lecture capture were predictive of performance. However, in that study only higher achieving students were able to use recorded lectures to overcome the negative impact of low attendance. This indicates that lecture attendance is probably not critical for highly engaged students with a high degree of self‐regulation [49] who catch up quickly and stay on top of things [50]. However, when we asked students when they normally watched recordings of missed lectures, only one‐third were likely to catch up within 1 week. By comparison, Euzent et al. [14] found that just over half of the students watched videos in the same week as the lecture, and 15% would normally watch videos for the first time close to the exam. What is more, our results show that students with good (self‐reported) attendance patterns were significantly more likely to catch up missed lectures quickly compared to students with low attendance who tended to delay watching the recordings. Although these correlations are not proof of causality, these findings suggest that lecture capture may encourage some students to skip class, and that the same students are more likely to use last‐minute cramming, a strategy that is not likely to be effective [51]. According to Stroup et al. [21] lecture capture can be an effective tool to make up missed lectures, but this depends very much on how the technology is used. Less committed students may be lured into thinking they can make up their many missed lectures by watching them later online, creating a false sense of security.

Another consideration in relation to study behaviour is how students make use of lecture recordings. In our study, there was a strong indication from both questionnaire and focus groups that many students use the recordings to create detailed notes, often attempting to write down everything that was said by the lecturer. This was frequently mentioned when students were asked to describe how they use lecture recordings but also in what way the recordings help them learn. They clearly place immense importance on having detailed notes, which is why they much appreciate being able to pause or slow down the video. Before the introduction of lecture capture, students would take notes during live lectures, but these notes were unlikely to be verbatim as students usually cannot write fast enough. Instead, students had to process the information and write down only the most important points. Jansen et al. [52] describe note taking as a multi‐stage process and the associated cognitive load may be too high in high‐speed, complex lectures. The authors suggest that the benefit of note taking can vary with students' cognitive ability and the benefit of note taking may be highest for students with high abilities. They conclude that the ability to pause and rewind lecture recordings may help to make note taking beneficial to all students regardless of their cognitive abilities. On the other hand, Mueller and Oppenheimer [53] claim that taking verbatim notes does not involve the processing of information and may therefore be detrimental to effective learning. The desire to create an almost word‐perfect transcript of the lecture reflects a passive learning approach associated with memorisation of lecture content rather than deep learning.

Using lecture recordings for the purpose of creating detailed notes is time intensive. Many students in our study reported spending a considerable amount of time watching recordings, even after attending class. A large percentage of respondents claimed to watch many lecture recordings from start to finish, sometimes multiple times. As a consequence, many students feel they do not have time to read textbooks or other materials on top of watching and note taking. What is more, in terms of material used for independent study, students prefer lecture recordings over textbooks or other sources. Indeed, some students told us that their revision is entirely based on watching lecture recordings. Similarly, Reid et al. [54] identified a tendency to rely on the ‘gospel word of the lecturer’. The authors found that a number of students were highly dependent on lecture capture and limited their learning to the use of lecture capture, believing that all the information they need would be in the recordings. Hockings et al. [55] identified independent learning as a key feature of higher education. This includes taking responsibility for their own learning, relying on self‐motivation and using multiple sources. Limiting their studies to writing lecture notes and watching recordings rather than searching and selecting information from different sources, students are implementing low‐level types of independent learning activities [55]. It is clear that extensive watching of lecture recordings does not equate to successful learning. Trenholm et al. [56] suggest that a reduction in lecture attendance coupled with reliance on lecture capture enables students to develop more surface approaches to learning. Similarly, Leadbeater et al. [17] found that students classified as high lecture capture users tended towards surface learning attitudes. These findings are mirrored by Reid et al. [54] who observed that students reporting a lower usage of lecture recordings were more likely to describe deep, active learning approaches.

In our study, we found that study patterns may change over time. Second‐year students reported significantly lower attendance compared with other year groups, while first‐year students' attendance was least affected by lecture capture. In terms of lecture capture usage, while first‐ and second‐year students tended to feel compelled to watch everything at least once, third‐year students tended to limit their viewing to specific lectures or parts of lectures. This may indicate an increase in self‐regulation and a more mature approach to study over time. Hockings et al. [55] suggest that first‐year students rely on their secondary school/college homework experience for their understanding of independent learning. Here, homework was described as being more tightly structured and was referred to as ‘spoon‐feeding’. Therefore, it may take some time for students adjusting to more appropriate approaches to independent learning in higher education. However, our study also indicates that usage of lecture capture versus (or in addition to) other material is partly assessment‐driven: As assessments change towards longer pieces of writing with the expectation of using outside reading in higher years, this may also help explain the changing patterns of lecture capture viewing across years of study.

Finally, a number of participants in our study mention a positive impact of lecture capture on their well‐being. Students report a reduction in stress and worry in relation to missing lectures due to illness or disabilities, mirroring findings by Williams and Fardon [57]. They also feel they can enjoy lectures more without having to worry about missing notes or slipping attention. The flip side is that students get anxious if a lecturer forgets to record, or there are technical problems with the recordings. Students feel dependent on lecture recordings, reporting a sense of stress and panic at the thought of not having them. This dependence on lecture capture was also observed by Reid et al. [54] who found that many students relied entirely on recorded lectures.

This study has a number of limitations. Firstly, it is based on perceptions and self‐reported behaviour (e.g. class attendance rates and viewing behaviour). Secondly, our participants were students in biological sciences, and our findings may not extend to other disciplines. Thirdly, our participants were self‐selected and may not be representative of the whole cohort. Even though more than half of the cohort responded to our survey, it is likely that the participants consisted of students who were reasonably well‐engaged with their studies. Other participant characteristics may also differ from the whole cohort, such as ease of internet access and other study conditions. Also, the number of focus group participants was relatively small and they were unlikely to be representative of the whole cohort. And finally, it is important to stress that in the context of this study, the term ‘lecture’ refers to traditional, mainly didactic lectures, and the recordings consisted of lecture slides with the real‐time recording of the lecturer's voice. An in‐depth discussion of the worth of didactic lectures versus other teaching formats is beyond the scope of this paper (for a reflection on this topic see for example [58]).

Finally, it is important to note that this study was undertaken at a time when live lectures were the standard teaching approach, and lecture capture was meant to complement rather than replace live lectures. As a consequence of the Covid‐19 pandemic, most or all classes in the UK went online from March 2020 and blended learning was widely used. Robson et al. [59] found that during the pandemic lectures were widely replaced by a mixture of synchronous and asynchronous classes, with most synchronous sessions being adapted to foster a more active learning approach. These types of teaching approaches (using a flipped classroom approach) may yield results that are different from our study [e.g. 60]. However, although Robson et al. [59] found that most academics in their study would prefer not to return to traditional lecturing, almost 40% believed we would return to pre‐pandemic teaching.

## Conclusions and recommendations

Lecture capture is clearly hugely popular among students, and many feel that they need the recordings in order to perform well in their exams. Our study suggests that for the majority of students, lecture capture does not appear to affect attendance. However, our findings suggest that lecture capture may contribute to some students' perception of learning as memorisation of the lecturer's spoken word. It may thereby reinforce their perceived dependency on a lecture transcript via detailed notes and foster surface learning behaviour. We also suggest that lecture capture could encourage a small group of students' unhelpful study habits including skipping class, procrastination and last‐minute cramming. Further studies are required to establish whether or not lecture capture is the actual cause of these study behaviours.

Whether or not institutions (or individuals) return to didactic lecturing in the postpandemic teaching environment, based on the results of our study we have two recommendations: Firstly, to provide guidance to students (and staff) on the effective use of lecture recordings [see e.g. 43]; and secondly, to adopt teaching and assessment strategies that do not rely entirely on memorisation of lecture material.

## Conflict of interest

The authors declare no conflict of interest.

### Peer review

The peer review history for this article is available at https://publons.com/publon/10.1002/2211‐5463.13548.

### Author contributions

SV and LVM conceived the project. SV, AB, TG, CL, ES, GW and LM designed the project, acquired, analysed and interpreted the data, and SV wrote the paper.

## Data Availability

All data not subject to limited distribution due to confidentiality agreements are available upon reasonable request to the first author, SV.

## References

[1] Ibrahim Y , Howarth A , Stone I . Lecture capture policies: a survey of British universities. Postdigit Sci Educ. 2021;3:144–61. 10.1007/s42438-020-00102-x

[2] Panopto . 75 studies reveal the impact of lecture capture. 2020 [cited 2021 Dec 30]. Available from: https://www.panopto.com/blog/75‐studies‐reveal‐the‐impact‐of‐lecture‐capture/

[3] O'Callaghan FV , Neumann DL , Jones L , Creed PA . The use of lecture recordings in higher education: a review of institutional, student and lecturer issues. Educ Inf Technol (Dordr). 2017;22:399–415. 10.1007/s10639-015-9451-z

[4] Gosper M , Green D , McNeill M , Woo K , Phillips R , Preston G . Final report: the impact of web‐based lecture technologies on current and future practices in learning and teaching. Sydney, NSW: Australian Learning and Teaching Council; 2008.

[5] Banerjee S . To capture the research landscape of lecture capture in university education. Comput Educ. 2021;160:104032. 10.1016/j.compedu.2020.104032 33020680 PMC7525658

[6] Dommett EJ , Gardner B , van Tilburg W . Staff and student views of lecture capture: a qualitative study. Int J Educ Technol High Educ. 2019;16(23):1–12. 10.1186/s41239-019-0153-2 PMC9038223 35496323

[7] Chapin LA . Australian university students' access to web‐based lecture recordings and the relationship with lecture attendance and academic performance. Australas J Educ Technol. 2018;34(5):1–12. 10.14742/ajet.2989

[8] Gorissen P , van Bruggen J , Jochems W . Students and recorded lectures: survey on current use and demands for higher education. Res Learn Technol. 2012;20:297–311. 10.3402/rlt.v20i0.17299

[9] Shaw GP , Molnar D . Non‐native English language speakers benefit most from the use of lecture capture in medical school. Biochem Mol Biol Educ. 2011;39(6):416–20. 10.1002/bmb.20552 22081545

[10] Nightingale KP , Anderson V , Onens S , Fazil Q , Davies H . Developing the inclusive curriculum: is supplementary lecture recording an effective approach in supporting students with specific learning difficulties (SpLDs)? Comput Educ. 2019;130:13–25. 10.1016/j.compedu.2018.11.006

[11] Nkomo LM , Daniel BK . Sentiment analysis of student engagement with lecture recording. TechTrends. 2021;65:213–24. 10.1007/s11528-020-00563-8 33432309 PMC7787655

[12] Morris NP , Swinnerton B , Coop T . Lecture recordings to support learning: a contested space between students and teachers. Comput Educ. 2019;140:103604. 10.1016/j.compedu.2019.103604

[13] Parson V , Reddy P , Wood J , Senior C . Educating an iPod generation: undergraduate attitudes, experiences and understanding of vodcast and podcast use. Learn Media Technol. 2009;34(3):215–28.

[14] Euzent P , Martin T , Moskal P , Moskal P . Assessing student performance and perceptions in lecture capture vs. face‐to‐face course delivery. J Inform Technol Educ. 2011;10:295–307.

[15] Gosper M , McNeill M , Woo K , Phillips R , Preston G , Green D . Web‐based lecture technologies and learning and teaching: a study of change in four Australian universities. JALN. 2010;15(4):84–95.

[16] Hall G , Ivaldi A . A qualitative approach to understanding the role of lecture capture in student learning experiences. Technol Pedagogy Educ. 2017;26(4):383–94. 10.1080/1475939X.2016.1263805

[17] Leadbeater W , Shuttleworth T , Couperthwaite J , Nightingale KP . Evaluating the use and impact of lecture recording in undergraduates: evidence for distinct approaches by different groups of students. Comput Educ. 2013;61:185–92.

[18] Smith K , Morris NP . Evaluation of biomedical science students use and perceptions of podcasting. Biosci Educ. 2014;22(1):3–15.

[19] Al Nashash H , Gunn C . Lecture capture in engineering classes: bridging gaps and enhancing learning. Educ Technol Soc. 2013;16(1):69–78.

[20] Groen JF , Quigley B , Herry Y . Examining the use of lecture capture technology: implications for teaching and learning. Can J Scholarsh Teach Learn. 2016;7(1). 10.5206/cjsotl-rcacea.2016.1.8

[21] Stroup MD , Pickard MM , Kahler KE . Testing the effectiveness of lecture capture technology using prior GPA as a performance indicator, *teacher‐scholar* . J State Comprehensive Univ. 2012;4(1):43–54.

[22] Lancaster JW , Wong A , Roberts SJ . 'Tech' versus 'talk': a comparison study of two different lecture styles within a master of science nurse practitioner course. Nurse Educ Today. 2012;32:e14–8. 22071277 10.1016/j.nedt.2011.09.018

[23] Morris C , Chikwa G . Screencasts: how effective are they and how do students engage with them? Active Learn High Educ. 2014;15(1):25–37.

[24] Brotherton JA , Abowd GD . Lessons learned from eClass: assessing automated capture and access in the classroom. ACM Trans Comput Hum Interact. 2004;11(2):121–55.

[25] Owston R , Lupshenyuk D , Wideman H . Lecture capture in large undergraduate classes: student perceptions and academic performance. Internet High Educ. 2011;14:262–8.

[26] Johnston ANB , Massa H , Burne THJ . Digital lecture recording: a cautionary tale. Nurse Educ Pract. 2013;13:40–7. 22889680 10.1016/j.nepr.2012.07.004

[27] Witchel HJ , Guy R , Torrens C , Langlands K , Doggrell SA . Chapter 12: attendance debate part 2. Lecture capture, attendance, and exam performance in the biosciences: exploring rare exceptions to the link between attendance and performance in the era of online teaching. In: Witchel HJ , Lee W , editors. Technologies in Biomedical and Life Sciences Education, methods in physiology. Cham, Switzerland: Springer Nature Switzerland AG; 2022. p. 343–82.

[28] Artz B , Johnson M , Robson D , Siemers S . Live or lecture capture: evidence from a classroom random control trial. Int Rev Econ Educ. 2022;40:100240. 10.1016/j.iree.2022.100240

[29] Joseph‐Richard P , Jessop T , Okafor G , Almpanis T , Price D . Big brother or harbinger of best practice: can lecture capture actually improve teaching? Br Educ Res J. 2018;44(3):377–92.

[30] Bond S , Grussendorf S . Staff attitudes to lecture capture. London, UK: The London School of Economics and Political Science; 2013.

[31] Chang S . Academic perceptions of the use of Lectopia: a University of Melbourne example. In: Atkinson RJ , McBeath C , editors. ICT: providing choices for learners and learning. Proceedings ASCILITE Singapore 2007. Singapore: Centre for Educational Development, Nanyang Technological University; 2007. p. 135–44.

[32] Dommett EJ , Gardner B , van Tilburg W . Staff and student perception of lecture capture. Internet High Educ. 2020;46:100732. 10.1016/j.iheduc.2020.100732

[33] Maynor LM , Landis Barrickman A , Stamatakis MK , Elliott DP . Student and faculty perceptions of lecture recordings in a doctor of pharmacy curriculum. Am J Pharm Educ. 2013;77(8):165. 24159206 10.5688/ajpe778165 PMC3806949

[34] Chen J , Lin T‐F . Do supplemental online recorded lectures help students learn microeconomics? Int Rev Econ Educ. 2021;11(1):6–15.

[35] McLean JL , Suchman EL . Video lecture capture technology helps students study without affecting attendance in large microbiology lecture courses. J Microbiol Biol Educ. 2016;17(3):480–1. 10.1128/jmbe.v17i3.1123 28101280 PMC5134957

[36] Edwards MR , Clinton ME . A study exploring the impact of lecture capture availability and lecture capture usage on student attendance and attainment. High Educ. 2019;77:403–21. 10.1007/s10734-018-0275-9

[37] Fernandes L , Maley M , Cruickshanks C . The impact of online lecture recordings on learning outcomes in pharmacology. J Int Assoc Med Sci Educ. 2008;18(2):62–70.

[38] Traphagan T , Kucsera JV , Kishi K . Impact of class lecture webcasting on attendance and learning. Educ Technol Res Dev. 2010;58(1):19–37. 10.1007/s11423-009-9128-7

[39] Chen J , Lin T‐F . Class attendance and exam performance: a randomized experiment. J Econ Educ. 2008;39(3):213–27.

[40] Dollinger SJ , Matyja AM , Huber JL . Which factors best account for academic success: those which students can control or those they cannot? J Res Pers. 2008;42:872–85.

[41] Newman‐Ford L , Fitzgibbon K , Lloyd S , Thomas S . A large‐scale investigation into the relationship between attendance and attainment: a study using an innovative, electronic attendance monitoring system. Stud High Educ. 2008;33(6):699–717.

[42] Williams A , Birch E , Hancock P . The impact of online lecture recordings on student performance. Australas J Educ Technol. 2012;28(2):199–213.

[43] Nordmann E , Kuepper‐Tetzel CE , Robson L , Phillipson S , Lipan GI , McGeorge P . Lecture capture: practical recommendations for students and instructors. Scholarsh Teach Learn Psychol. 2020;8:174–93. 10.1037/stl0000190

[44] Horn D . Recorded lectures are not for everyone: lower‐performing students benefit from attending live lectures. Optom Educ. 2020;46(1):1–9.

[45] Creswell JW , Plano Clark VL . Designing and conducting mixed method research. 3rd ed. Los Angeles, CA, SAGE Publications, Inc; 2018.

[46] Braun V , Clarke V . Using thematic analysis in psychology. Qual Res Psychol. 2006;3(2):77–101.

[47] Holbrook J , Dupont C . Profcasts and class attendance — does year in program matter? Biosci Educ. 2009;13(1):1–4. 10.3108/beej.13.c2

[48] Franklin DS , Gibson JW , Samuel JC , Teeter WA , Clarkson CW . Use of lecture recordings in medical education. J Int Assoc Med Sci Educ. 2011;1:21–8.

[49] Richardson M , Abraham C , Bond R . Psychological correlates of university students' academic performance: a systematic review and meta‐analysis. Psychol Bull. 2012;138(2):353–87. 10.1037/a0026838 22352812

[50] Liles J , Vuk J , Tariq S . Study habits of medical students: an analysis of which study habits most contribute to success in the preclinical years [version 1]. MedEdPublish. 2018;7(61):1–19. 10.15694/mep.2018.0000061.1 PMC10712003 38089203

[51] Chai A , Guest R . Exploring the links between online lecture recordings, cramming and academic performance. Australas J Econ Educ. 2017;14(1):1–30.

[52] Jansen RS , Lakens D , Ijsselsteijn WA . An integrative review of the cognitive costs and benefits of not‐taking. Educ Res Rev. 2017;22:223–33. 10.1016/j.edurev.2017.10.001

[53] Mueller PA , Oppenheimer DM . The pen is mightier than the keyboard: advantages of longhand over laptop note taking. Psychol Sci. 2014;25(6):1159–68. 10.1177/0956797614524581 24760141

[54] Reid A , Duret D , Noble K . Lecture capture: friend or foe? J Vet Med Educ. 2021;49:126–37. 10.3138/jvme-2020-0052 33956584

[55] Hockings C , Thomas L , Ottaway J , Jones R . Independent learning – what we do when you're not there. Teach High Educ. 2018;23(2):145–61. 10.1080/13562517.2017.1332031

[56] Trenholm S , Hajek B , Robinson CL , Chinnappan M , Albrecht A , Ashman H . Investigating undergraduate mathematics learners' cognitive engagement with recorded lecture videos. Int J Math Educ Sci Technol. 2019;50(1):3–24. 10.1080/0020739X.2018.1458339

[57] Williams J , Fardon M . Lecture recordings: extending access for students with disabilities. In: Wheeler S , Whitton N , editors. ALT‐C 2007: beyond control, learning technology for the social network generation, research proceedings (Nottingham university EMCC*,* UK ed.), Vol. 1. Chesterton: The Association for Learning Technology; 2007. p. 139–48.

[58] Jones SE . Reflections on the lecture: outmoded medium or instrument of inspiration? J Further High Educ. 2007;31(4):397–406. 10.1080/03098770701656816

[59] Robson L , Gardner B , Dommett EJ . The post‐pandemic lecture: views from academic staff across the UK. Educ Sci. 2022;12:123. 10.3390/educsci12020123

[60] Yamarik S . Flipping the classroom and student learning outcomes: evidence from an international economics course. Int Rev Econ Educ. 2019;31:1–12. 10.1016/j.iree.2019.100163

